# Supplementary material for: Pleiotropic actions of phenothiazine drugs are detrimental to Gram-negative bacterial persister cells
Source: Commun Biol. 2022 Mar 9;5:217. doi: 10.1038/s42003-022-03172-8 (PMC8907348; doi:10.1038/s42003-022-03172-8)
Supplement: Supplementary file 2 — Supplementary Information [file 42003_2022_3172_MOESM2_ESM.pdf]

Supplementary Information

Pleiotropic actions of phenothiazine drugs are detrimental to Gram-negative bacterial persister cells

Sayed Golam Mohiuddin[1], Thao Vy Nguyen[2], and Mehmet A. Orman[3]*

[1,2,3]Department of Chemical and Biomolecular Engineering, University of Houston, Houston, TX, USA

*Correspondence to: S222 Engineering Bldg 1, 4726 Calhoun Rd, Houston, TX 77204, Phone: 713-743-6785, Email: morman@central.uh.edu

SUPPLEMENTARY METHOD

Metabolomics

The methodologies described below were provided by Metabolomics, Inc.

Sample Accessioning: Following receipt, samples were inventoried and immediately stored at -80 ºC. Each sample received was accessioned into the Metabolon LIMS system and was assigned by the LIMS a unique identifier that was associated with the original source identifier only. This identifier was used to track all sample handling, tasks, results, etc. The samples (and all derived aliquots) were tracked by the LIMS system. All portions of any sample were automatically assigned their own unique identifiers by the LIMS when a new task was created; the relationship of these samples was also tracked. All samples were maintained at -80 ºC until processed.

Sample Preparation: Samples were prepared using the automated MicroLab STAR® system from Hamilton Company. Several recovery standards were added prior to the first step in the extraction process for (quality control) QC purposes. To remove protein, dissociate small molecules bound to protein or trapped in the precipitated protein matrix, and to recover chemically diverse metabolites, proteins were precipitated with methanol under vigorous shaking for 2 min (Glen Mills Geno Grinder 2000) followed by centrifugation. The resulting extract was divided into five fractions: two for analysis by two separate reverse phases (RP)/UPLC-MS/MS methods with positive ion mode electrospray ionization (ESI), one for analysis by RP/UPLC-MS/MS with negative ion mode ESI, one for analysis by HILIC/UPLC-MS/MS with negative ion mode ESI, and one sample was reserved for backup. Samples were placed briefly on a TurboVap® (Zymark) to remove the organic solvent. The sample extracts were stored overnight under nitrogen before preparation for analysis.

QA/QC: Several types of controls were analyzed in concert with the experimental samples: a pooled matrix sample generated by taking a small volume of each experimental sample served as a technical replicate throughout the data set; extracted water samples served as process blanks; and a cocktail of QC standards that were carefully chosen not to interfere with the measurement of endogenous compounds were spiked into every analyzed sample, allowed instrument performance monitoring and aided chromatographic alignment. Instrument variability was determined by calculating the median relative standard deviation (RSD) for the standards that were added to each

sample prior to injection into the mass spectrometers. Overall process variability was determined by calculating the median RSD for all endogenous metabolites (i.e., non-instrument standards) present in 100% of the pooled matrix samples. Experimental samples were randomized across the platform run with QC samples spaced evenly among the injections.

Ultrahigh Performance Liquid Chromatography-Tandem Mass Spectroscopy (UPLC-MS/MS): All methods utilized a Waters ACQUITY ultra-performance liquid chromatography (UPLC) and a Thermo Scientific Q-Exactive high resolution/accurate mass spectrometer interfaced with a heated electrospray ionization (HESI-II) source and Orbitrap mass analyzer operated at 35,000 mass resolution. The sample extract was dried then reconstituted in solvents compatible to each of the four methods. Each reconstitution solvent contained a series of standards at fixed concentrations to ensure injection and chromatographic consistency. One aliquot was analyzed using acidic positive ion conditions, chromatographically optimized for more hydrophilic compounds. In this method, the extract was gradient eluted from a C18 column (Waters UPLC BEH C18-2.1x100 mm, 1.7 µm) using water and methanol, containing 0.05% perfluoropentanoic acid (PFPA) and 0.1% formic acid (FA). Another aliquot was also analyzed using acidic positive ion conditions; however, it was chromatographically optimized for more hydrophobic compounds. In this method, the extract was gradient eluted from the same afore mentioned C18 column using methanol, acetonitrile, water, 0.05% PFPA and 0.01% FA and was operated at an overall higher organic content. Another aliquot was analyzed using basic negative ion optimized conditions using a separate dedicated C18 column. The basic extracts were gradient eluted from the column using methanol and water, however with 6.5mM Ammonium Bicarbonate at pH 8. The fourth aliquot was analyzed via negative ionization following elution from a HILIC column (Waters UPLC BEH Amide 2.1x150 mm, 1.7 µm) using a gradient consisting of water and acetonitrile with 10 mM Ammonium Formate, pH 10.8. The MS analysis alternated between MS and data-dependent MSn scans using dynamic exclusion. The scan range varied slighted between methods but covered 70-1000 m/z. Raw data files are archived and extracted as described below.

Bioinformatics: The informatics system consisted of four major components, the Laboratory Information Management System (LIMS), the data extraction and peak-identification software, data processing tools for QC and compound identification, and a collection of information interpretation and visualization tools for use by data analysts. The hardware and software

foundations for these informatics components were the LAN backbone, and a database server running Oracle 10.2.0.1 Enterprise Edition.

LIMS: The purpose of the Metabolon LIMS system was to enable fully auditable laboratory automation through a secure, easy to use, and highly specialized system. The scope of the Metabolon LIMS system encompasses sample accessioning, sample preparation and instrumental analysis and reporting and advanced data analysis. All the subsequent software systems are grounded in the LIMS data structures. It has been modified to leverage and interface with the in-house information extraction and data visualization systems, as well as third party instrumentation and data analysis software.

Data Extraction and Compound Identification: Raw data was extracted, peak-identified and QC processed using Metabolon's hardware and software. These systems are built on a web-service platform utilizing Microsoft's .NET technologies, which run on high-performance application servers and fiber-channel storage arrays in clusters to provide active failover and load-balancing. Compounds were identified by comparison to library entries of purified standards or recurrent unknown entities. Metabolon maintains a library based on authenticated standards that contains the retention time/index (RI), mass to charge ratio (m/z), and chromatographic data (including MS/MS spectral data) on all molecules present in the library. Furthermore, biochemical identifications are based on three criteria: retention index within a narrow RI window of the proposed identification, accurate mass match to the library +/- 10 ppm, and the MS/MS forward and reverse scores between the experimental data and authentic standards. The MS/MS scores are based on a comparison of the ions present in the experimental spectrum to the ions present in the library spectrum. While there may be similarities between these molecules based on one of these factors, the use of all three data points can be utilized to distinguish and differentiate biochemicals. More than 3300 commercially available purified standard compounds have been acquired and registered into LIMS for analysis on all platforms for determination of their analytical characteristics. Additional mass spectral entries have been created for structurally unnamed biochemicals, which have been identified by virtue of their recurrent nature (both chromatographic and mass spectral). These compounds have the potential to be identified by future acquisition of a matching purified standard or by classical structural analysis.

Curation: A variety of curation procedures were carried out to ensure that a high-quality data set

was made available for statistical analysis and data interpretation. The QC and curation processes were designed to ensure accurate and consistent identification of true chemical entities, and to remove those representing system artifacts, mis-assignments, and background noise. Metabolon data analysts use proprietary visualization and interpretation software to confirm the consistency of peak identification among the various samples. Library matches for each compound were checked for each sample and corrected if necessary.

Metabolite Quantification and Data Normalization: Peaks were quantified using area-under-the-curve. For studies spanning multiple days, a data normalization step was performed to correct variation resulting from instrument inter-day tuning differences. Essentially, each compound was corrected in run-day blocks by registering the medians to equal one (1.00) and normalizing each data point proportionately. For studies that did not require more than one day of analysis, no normalization is necessary, other than for purposes of data visualization. In certain instances, biochemical data may have been normalized to an additional factor (e.g., cell counts, total protein as determined by Bradford assay, osmolality, etc.) to account for differences in metabolite levels due to differences in the amount of material present in each sample.

SUPPLEMENTARY FIGURES

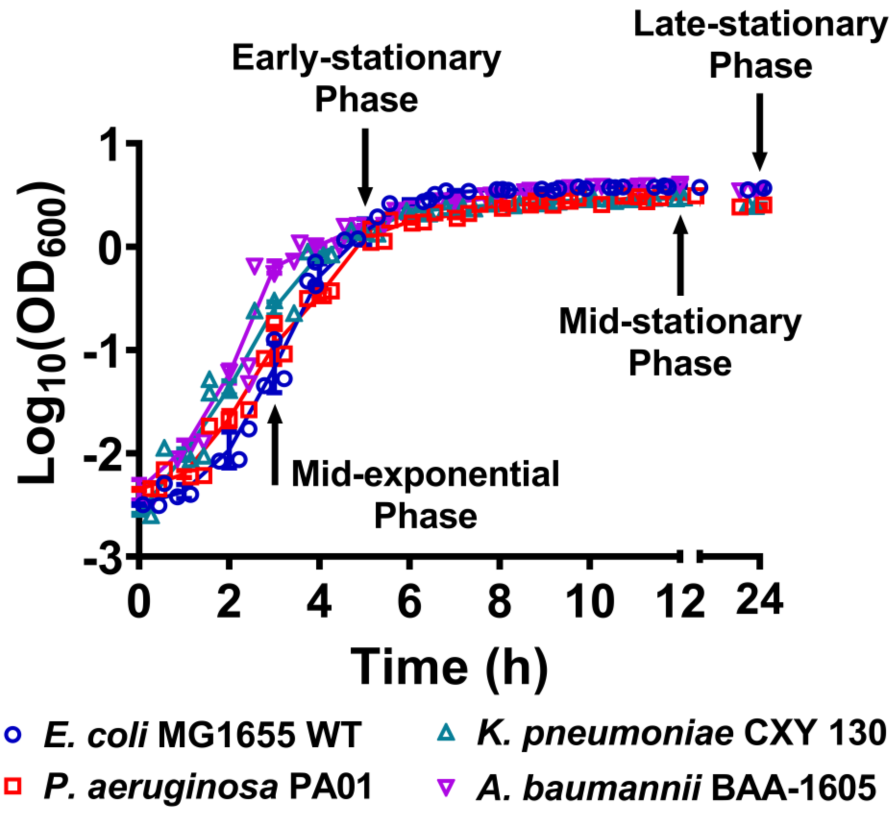

Supplementary Fig. 1. Growth curves of *E. coli* MG1655 WT, *P. aeruginosa* PA01, *K. pneumoniae* CXY 130 and *A. baumannii* BAA-1605. Overnight cultures were diluted (1:1000) in fresh 25-ml LB (for *E. coli*, *K. pneumoniae* and *A. baumannii*) or MH (for *P. aeruginosa*) medium in 250-ml flasks and cultured at 37ºC and 250 rpm. At indicated time points, samples were collected for optical density (OD_{600}) measurements. Cell cultures generally reach the mid-exponential phase after about 3 h and early stationary phase after about 5 h. Cultures at t=12 h and t=24 h are considered mid- and late-stationary phase cultures, respectively. Number of independent biological replicates, n=3. Data corresponding to each time point represent mean value ± standard deviation.

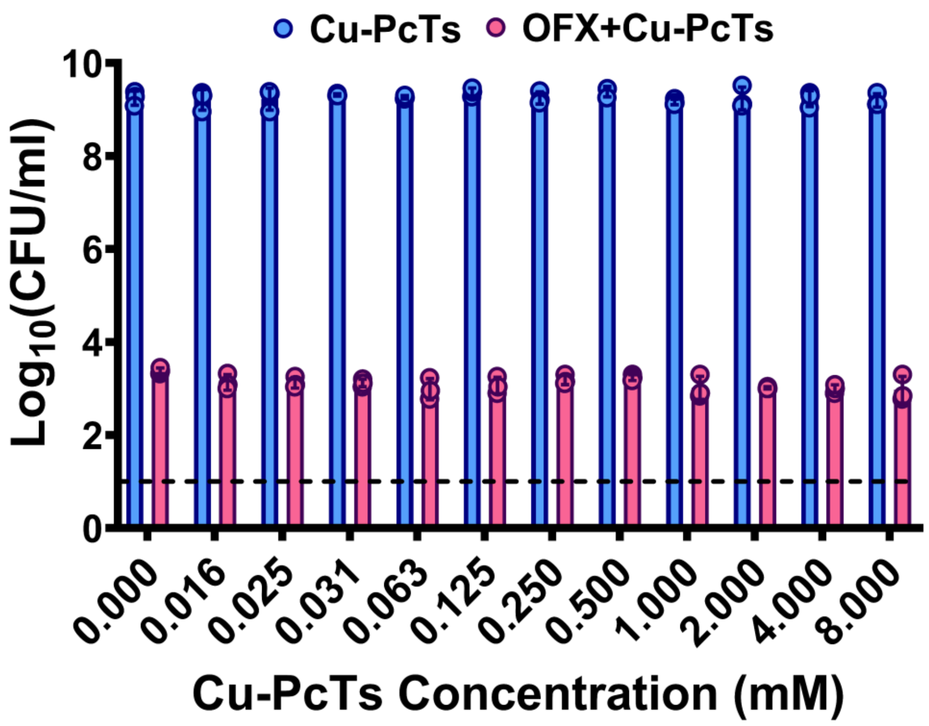

Supplementary Fig. 2. Cu-PcTs treatment does not reduce OFX persisters. Cells at early stationary phase (t=5 h) were treated with OFX (5 µg/ml) and Cu-PcTs (RecA inhibitor) at indicated concentrations for 20 h, and then, plated for viable cell counts. Dashed lines represent the limit of detection. n=3. Data corresponding to each time point represent mean value ± standard deviation.

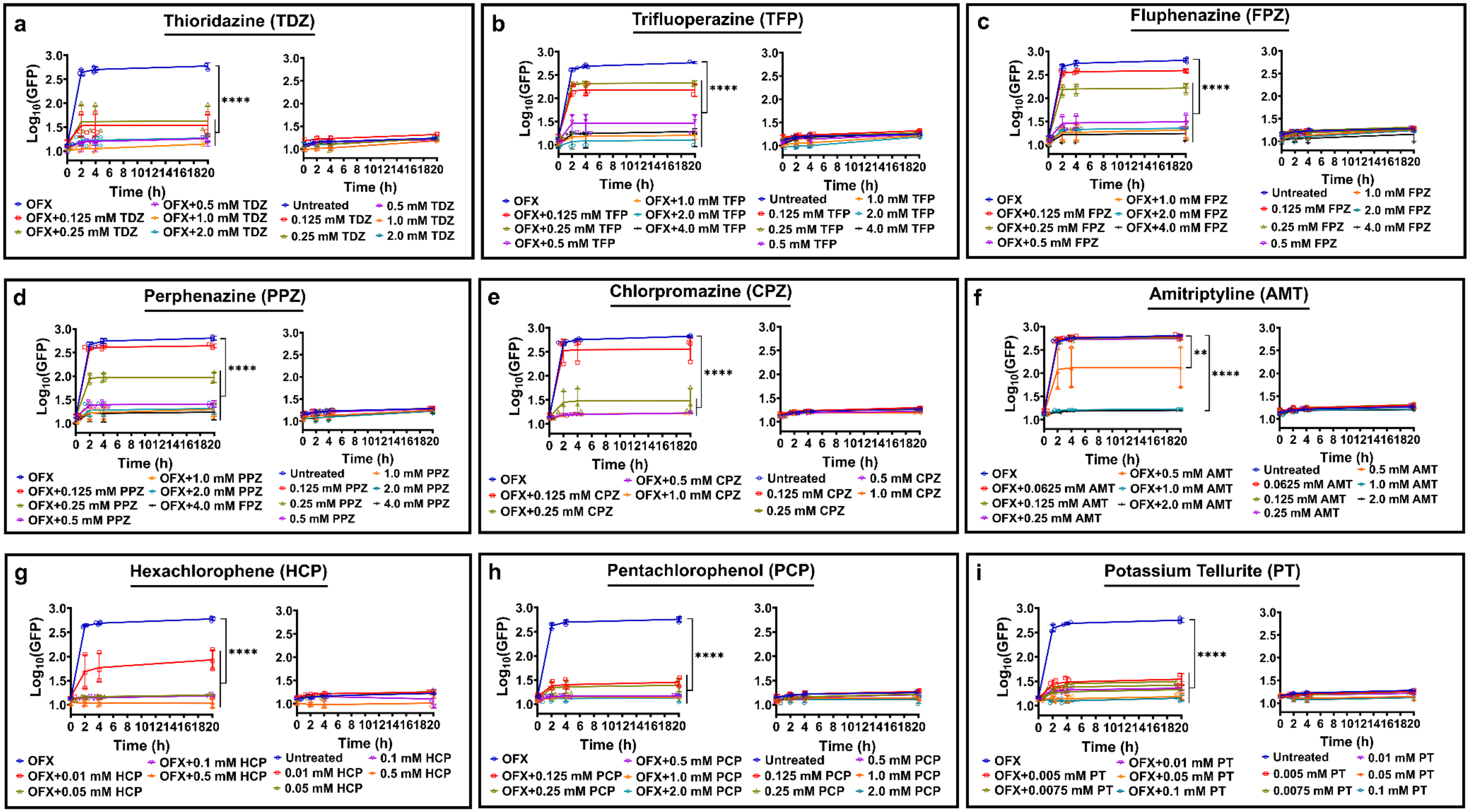
a
Thioridazine (TDZ)
Log10(GFP)
Time (h)
****
OFX
OFX+0.125 mM TDZ
OFX+0.25 mM TDZ
OFX+0.5 mM TDZ
OFX+1.0 mM TDZ
OFX+2.0 mM TDZ
Untreated
0.125 mM TDZ
0.25 mM TDZ
0.5 mM TDZ
1.0 mM TDZ
2.0 mM TDZ
b
Trifluoperazine (TFP)
OFX
OFX+0.125 mM TFP
OFX+0.25 mM TFP
OFX+0.5 mM TFP
OFX+1.0 mM TFP
OFX+2.0 mM TFP
OFX+4.0 mM TFP
Untreated
0.125 mM TFP
0.25 mM TFP
0.5 mM TFP
1.0 mM TFP
2.0 mM TFP
4.0 mM TFP
c
Fluphenazine (FPZ)
OFX
OFX+0.125 mM FPZ
OFX+0.25 mM FPZ
OFX+0.5 mM FPZ
OFX+1.0 mM FPZ
OFX+2.0 mM FPZ
OFX+4.0 mM FPZ
Untreated
0.125 mM FPZ
0.25 mM FPZ
0.5 mM FPZ
1.0 mM FPZ
2.0 mM FPZ
4.0 mM FPZ
d
Perphenazine (PPZ)
OFX
OFX+0.125 mM PPZ
OFX+0.25 mM PPZ
OFX+0.5 mM PPZ
OFX+1.0 mM PPZ
OFX+2.0 mM PPZ
OFX+4.0 mM FPZ
Untreated
0.125 mM PPZ
0.25 mM PPZ
0.5 mM PPZ
1.0 mM PPZ
2.0 mM PPZ
4.0 mM PPZ
e
Chlorpromazine (CPZ)
OFX
OFX+0.125 mM CPZ
OFX+0.25 mM CPZ
OFX+0.5 mM CPZ
OFX+1.0 mM CPZ
Untreated
0.125 mM CPZ
0.25 mM CPZ
0.5 mM CPZ
1.0 mM CPZ
f
Amitriptyline (AMT)
**
****
OFX
OFX+0.0625 mM AMT
OFX+0.125 mM AMT
OFX+0.25 mM AMT
OFX+0.5 mM AMT
OFX+1.0 mM AMT
OFX+2.0 mM AMT
Untreated
0.0625 mM AMT
0.125 mM AMT
0.25 mM AMT
0.5 mM AMT
1.0 mM AMT
2.0 mM AMT
g
Hexachlorophene (HCP)
OFX
OFX+0.01 mM HCP
OFX+0.05 mM HCP
OFX+0.1 mM HCP
OFX+0.5 mM HCP
Untreated
0.01 mM HCP
0.05 mM HCP
0.1 mM HCP
0.5 mM HCP
h
Pentachlorophenol (PCP)
OFX
OFX+0.125 mM PCP
OFX+0.25 mM PCP
OFX+0.5 mM PCP
OFX+1.0 mM PCP
OFX+2.0 mM PCP
Untreated
0.125 mM PCP
0.25 mM PCP
0.5 mM PCP
1.0 mM PCP
2.0 mM PCP
i
Potassium Tellurite (PT)
OFX
OFX+0.005 mM PT
OFX+0.0075 mM PT
OFX+0.01 mM PT
OFX+0.05 mM PT
OFX+0.1 mM PT
Untreated
0.005 mM PT
0.0075 mM PT
0.01 mM PT
0.05 mM PT
0.1 mM PT

Supplementary Fig. 3. Candidate chemicals can inhibit the *recA* expression. (a-i) *E. coli* MG1655 cells harboring the *recA* reporter were treated with OFX (5 μg/ml) and/or candidate chemicals at indicated concentrations at early stationary phase. GFP was measured in the treatment cultures for the indicated time points. Cells treated only with OFX served as the positive control; untreated cells served as the negative control. Candidate chemicals in the absence of OFX do not induce *recA* expression. Statistical comparison was performed using one-way ANOVA with Dunnett's posttest. $^{**}P < 0.01$ and $^{****}P < 0.0001$. n=3. Data corresponding to each time point represent mean value ± standard deviation.

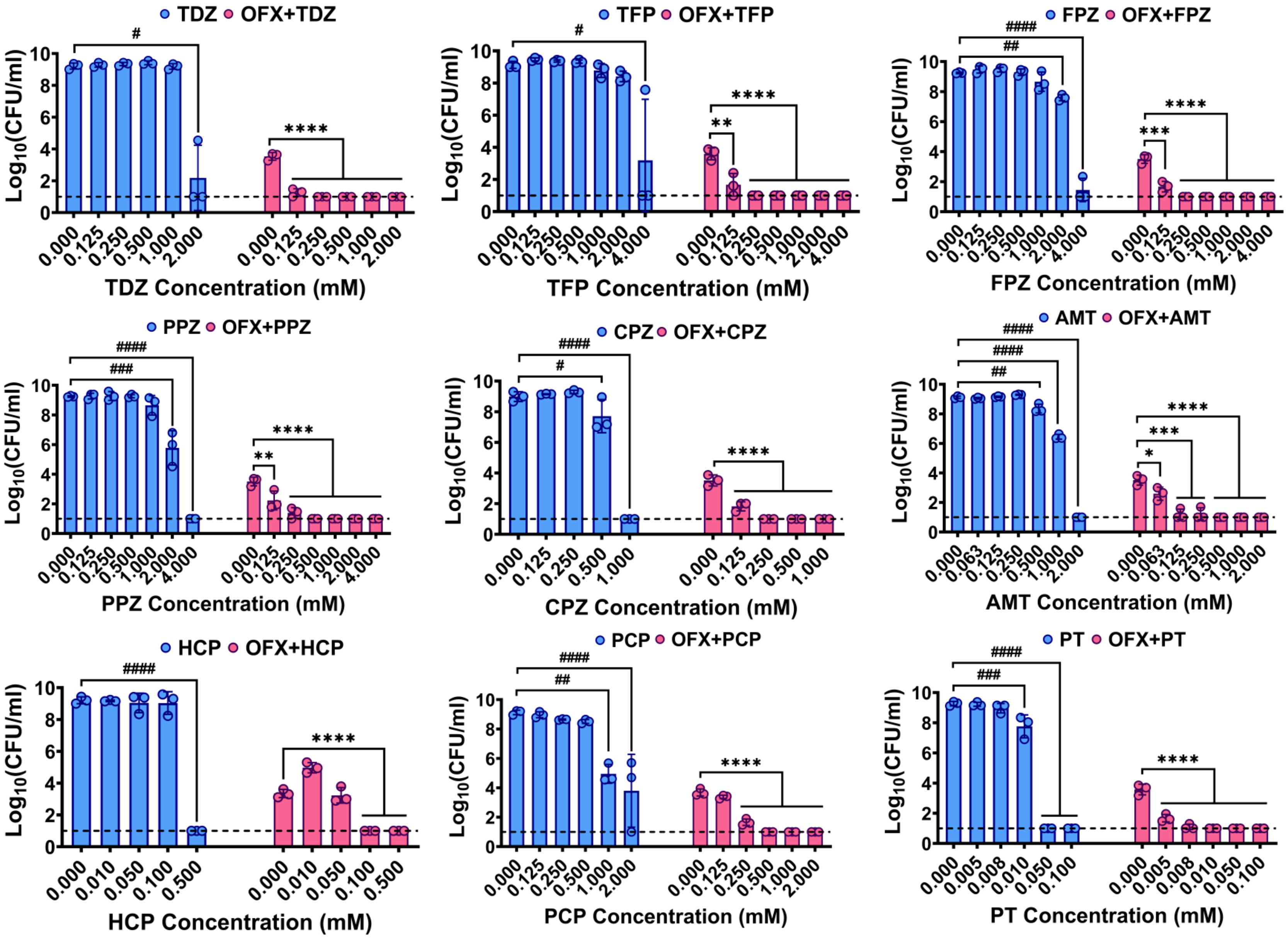
TDZ OFX+TDZ
Log10(CFU/ml)
TDZ Concentration (mM)
TFP OFX+TFP
TFP Concentration (mM)
FPZ OFX+FPZ
FPZ Concentration (mM)
PPZ OFX+PPZ
PPZ Concentration (mM)
CPZ OFX+CPZ
CPZ Concentration (mM)
AMT OFX+AMT
AMT Concentration (mM)
HCP OFX+HCP
HCP Concentration (mM)
PCP OFX+PCP
PCP Concentration (mM)
PT OFX+PT
PT Concentration (mM)

Supplementary Fig. 4. Candidate chemicals can reduce OFX persister levels. *E. coli* MG1655 cells, treated with OFX (5 µg/ml) and/or candidate chemicals for 20 h, were plated for viable cell counts. Dashed lines represent the limit of detection for CFU counts. Statistical analysis was performed using one-way ANOVA with Dunnett's posttest. $^{*\ \mathrm{or}\ \#}P < 0.05$, $^{**\ \mathrm{or}\ \#\#}P < 0.01$, $^{***\ \mathrm{or}\ \#\#\#}P < 0.001$, $^{****\ \mathrm{or}\ \#\#\#\#}P < 0.0001$. n=3. Data corresponding to each time point represent mean value ± standard deviation.

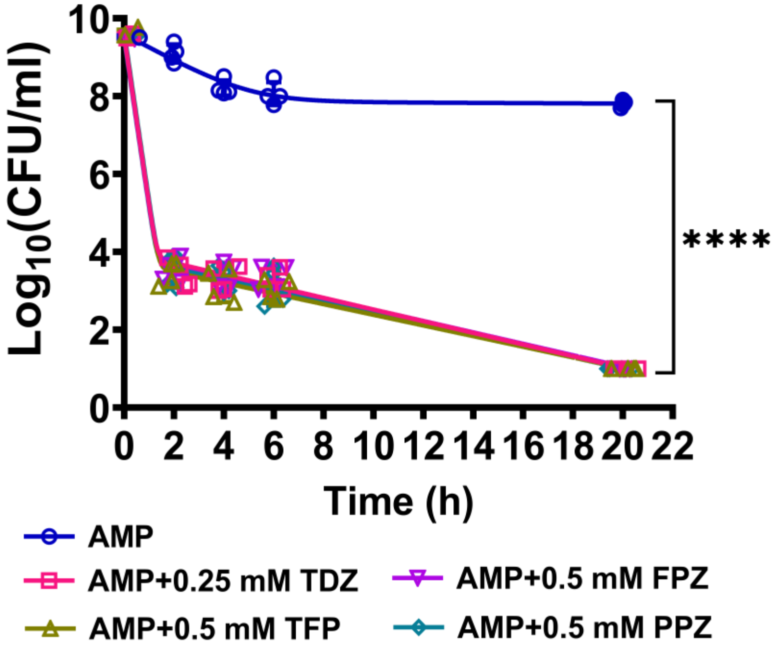

Supplementary Fig. 5. Phenothiazines reduce ampicillin persister levels. *E. coli* MG1655 cells were treated with ampicillin (200 µg/ml) and phenothiazines at indicated concentrations at early stationary phase. At indicated time points during the treatment, cells were plated for viable cell counts. The limit of viable cell detection is 10 CFUs/ml. Statistical analysis was performed using one-way ANOVA with Dunnett's posttest. ****P < 0.0001. n=4. Data corresponding to each time point represent mean value ± standard deviation.

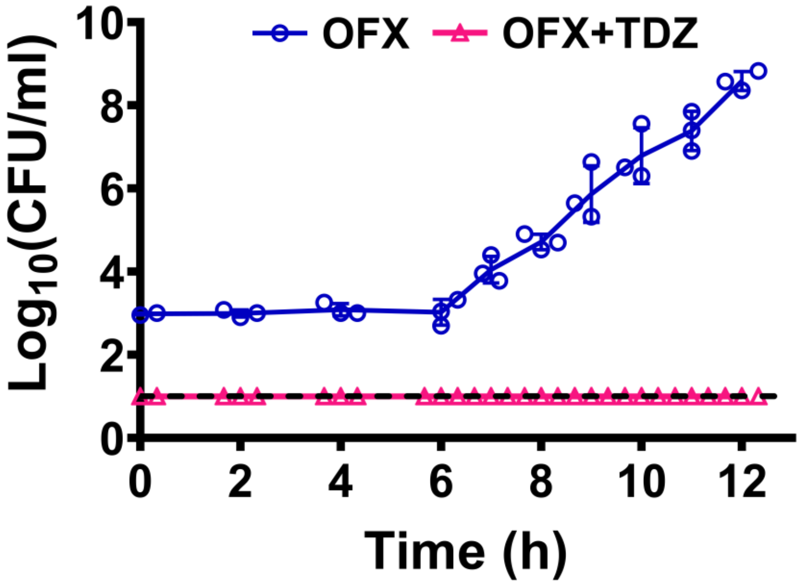

Supplementary Fig. 6. Growth curves during the recovery period. Early stationary phase cells (t=5 h) of *E. coli* MG1655 with P_{recA}-*gfp* were treated with OFX or OFX + TDZ for 20 h. After treatment, cells were washed, transferred to fresh liquid medium and cultured for persister recovery. The CFU measurement was performed during the recovery period. Persister levels of OFX+TDZ treated cultures are under the limit of detection (dashed line). n=3. Data corresponding to each time point represent mean value ± standard deviation.

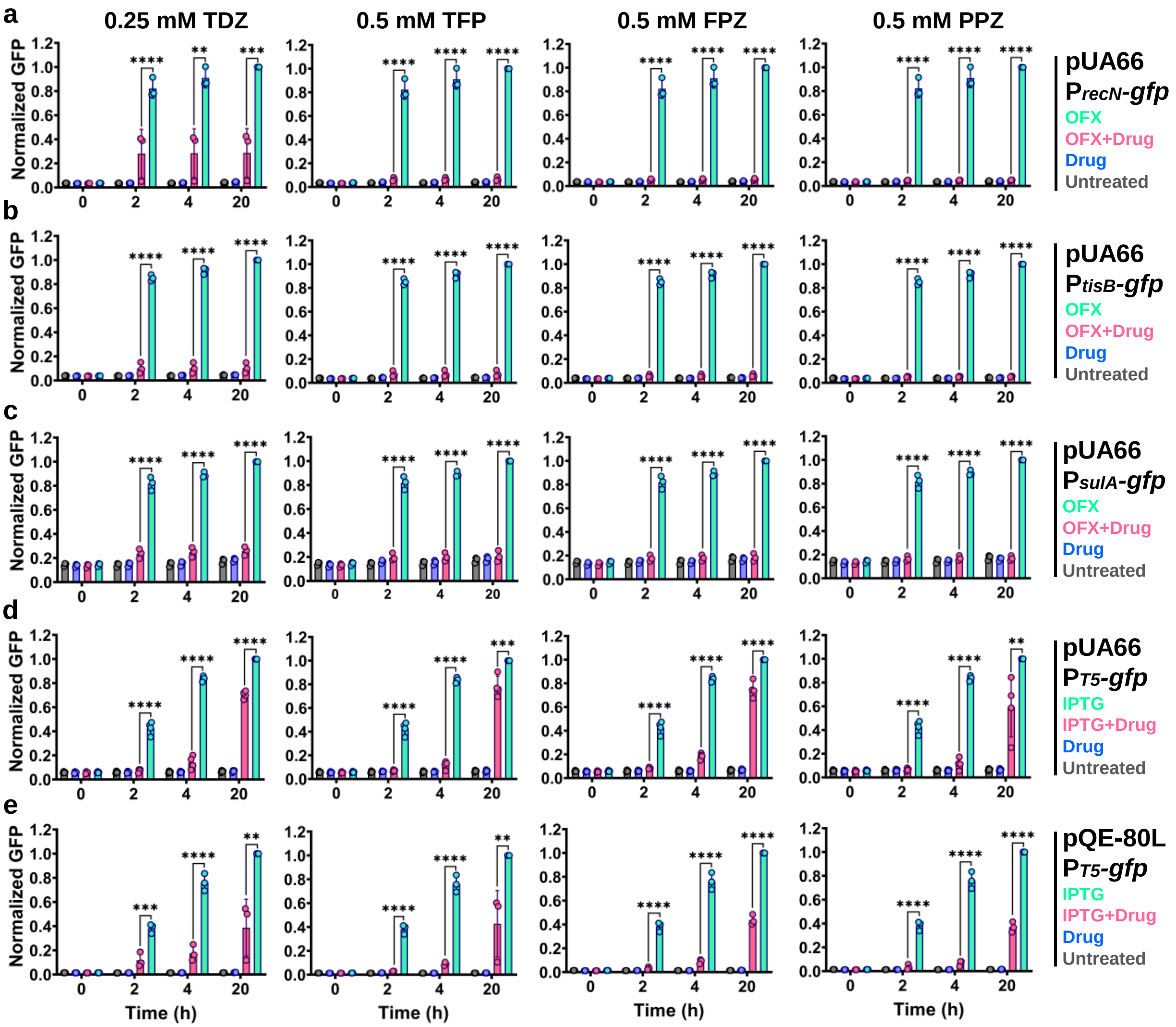

Supplementary Fig. 7. Phenothiazine drugs reduce the expression of SOS regulon genes by inhibiting transcription/translation in *E. coli* MG1655. (**a-c**) Inhibition of SOS gene expression by phenothiazine drugs. Cells with reporter plasmids were treated with OFX (5 µg/ml) or OFX + phenothiazine drugs at early stationary phase and GFP levels were measured with a plate reader. n=3. (**d-e**) Inhibition of transcription/translation by phenothiazine drugs. Cells with pUA66 and pQ-80L plasmids were treated with OFX (5 µg/ml) or OFX + phenothiazine drugs at early stationary phase and, at the same time, expression of GFP was induced with IPTG (1 mM). n=3. Statistical analysis was performed between the control and treatment groups using One-way ANOVA with Dunnett's posttest. **P < 0.01, ***P < 0.001, ****P < 0.0001. Data corresponding to each time point represent mean value ± standard deviation.

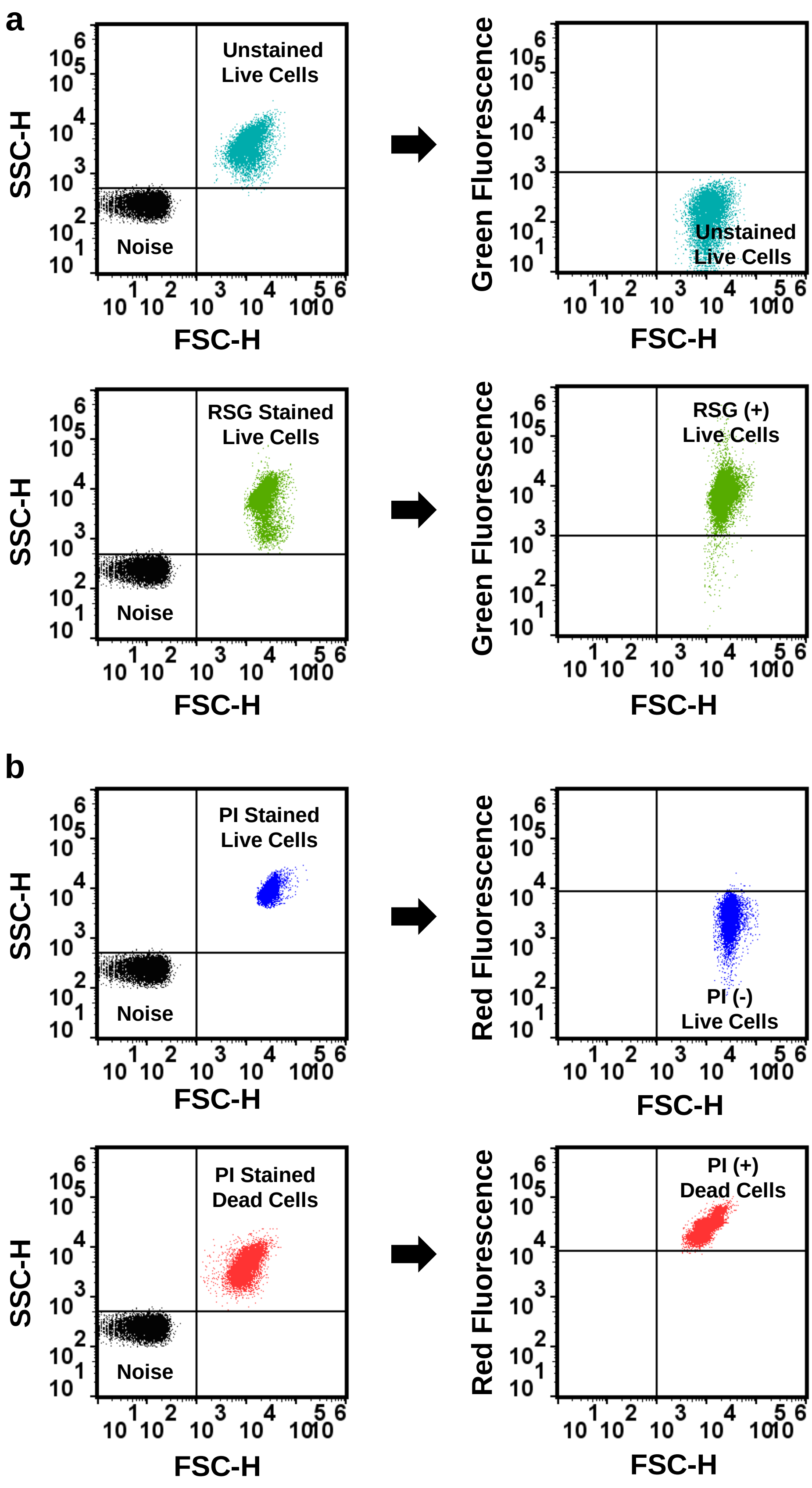
a
SSC-H
FSC-H
Unstained
Live Cells
Noise
Green Fluorescence
Unstained
Live Cells
RSG Stained
Live Cells
RSG (+)
Live Cells
b
PI Stained
Live Cells
Red Fluorescence
PI (-)
Live Cells
PI Stained
Dead Cells
PI (+)
Dead Cells

Supplementary Fig. 8. Flow cytometry gating strategy. Forward scatter (FSC-H) and side scatter (SSC-H) signals were used to distinguish cells and instrumental noises. A sterile PBS solution (without cells) was analyzed with the flow cytometer to determine the instrument noises. **(a)** Exponential phase cells were stained with RSG dye (1 µM) to gate the RSG (+) cell population on the flow diagram. Unstained cells were used to gate the RSG (-) cell population. A representative flow cytometry diagram is shown in the figure. All independent biological replicates have similar results. n=3. **(b)** Live cells and ethanol (70% v/v) treated dead cells were stained with PI to gate live [PI (-)] and dead [PI (+)] cell populations. A representative flow cytometry diagram is shown in the figure. All independent biological replicates have similar results. n=3. FSC-H: Forward Scatter.

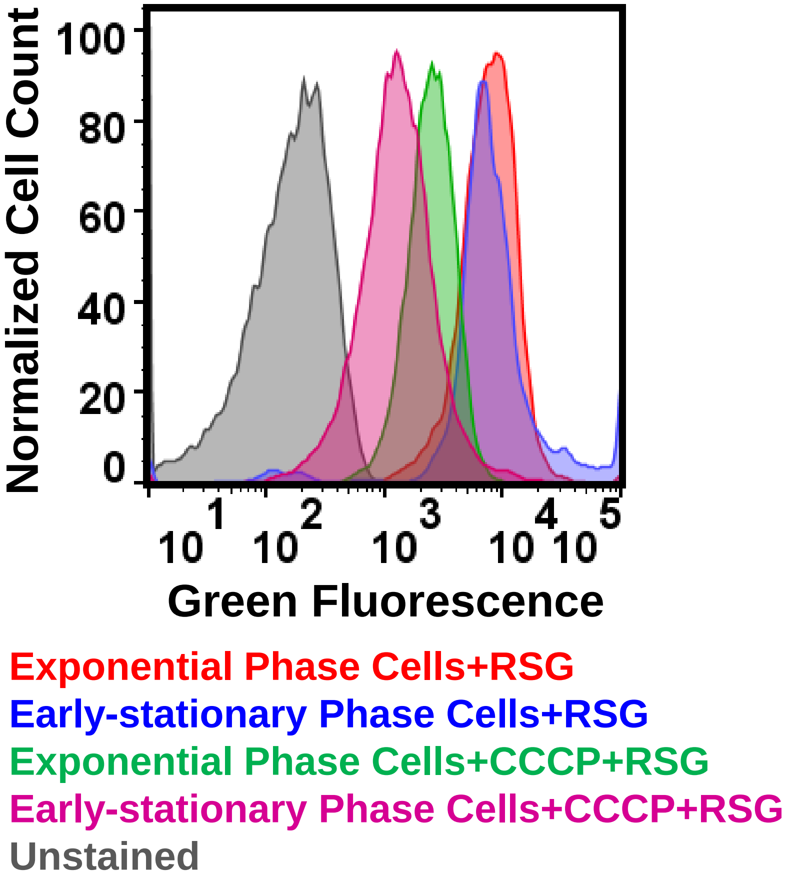

Supplementary Fig. 9. RSG staining to measure bacterial metabolic activities. Exponential and early stationary phase cells were stained with RSG (1 µM) prior to analysis by flow cytometry. Cells treated with CCCP (10 µM) + RSG (1 µM) served as a control. CCCP is expected to reduce cellular redox activities. A representative flow cytometry diagram is shown in the figure. All independent biological replicates have similar results. n=3.

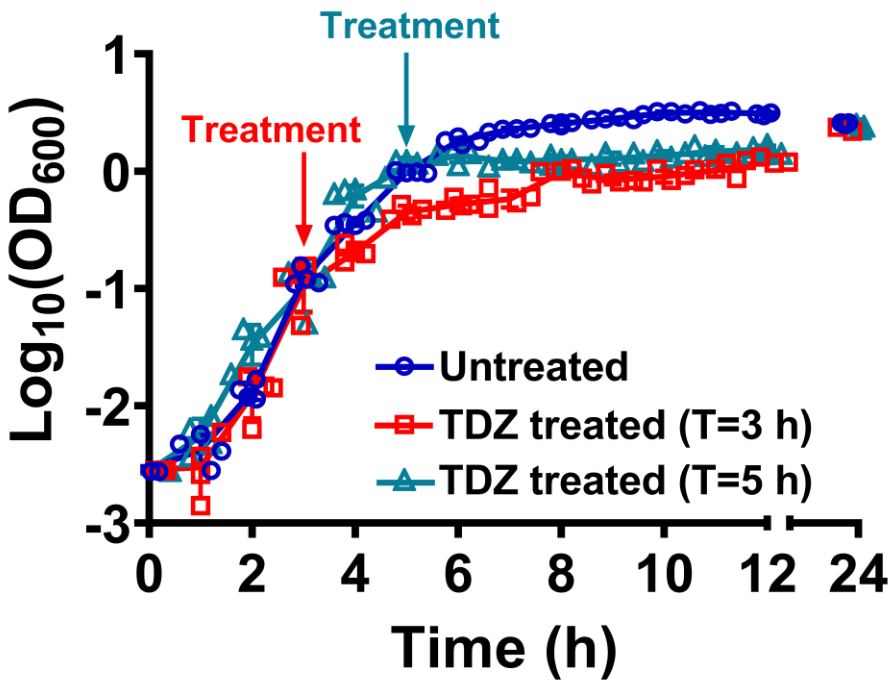

Supplementary Fig. 10. Growth curves of *E. coli* MG1655 cells in the presence of a phenothiazine drug. Overnight cultures were diluted (1:1000) in fresh 25-ml LB in 250-ml flasks and cultured at 37 ºC and 250 rpm. Cells were treated with thioridazine (TDZ) (0.25 mM) at t=3 h or t=5 h. At designated time points samples were collected for optical density (OD_{600}) measurements. n=4. Data corresponding to each time point represent mean value ± standard deviation.

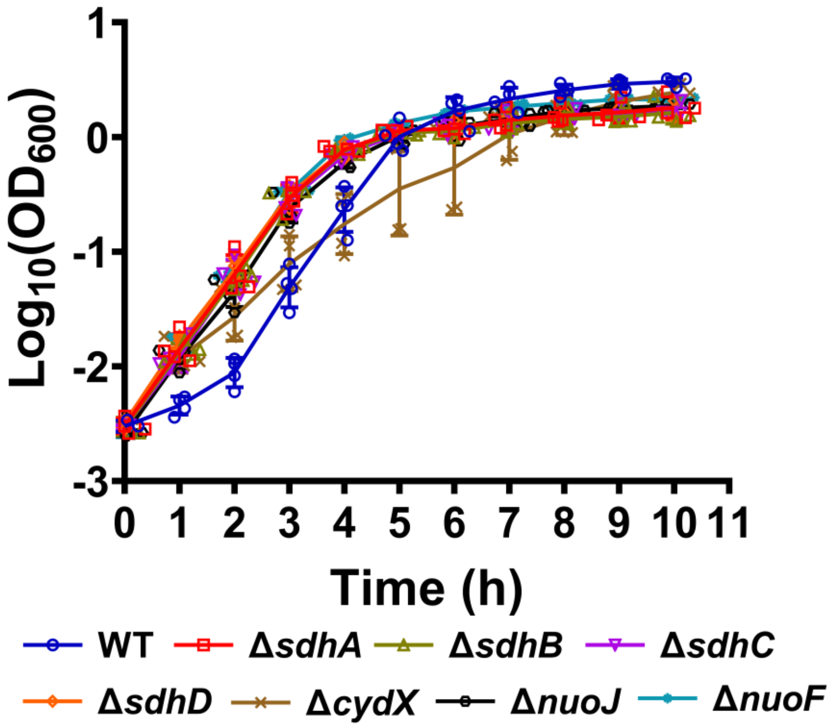

Supplementary Fig. 11. Growth curves of *E. coli* MG1655 single-deletion strains. Overnight cultures were diluted (1:1000) in fresh 2-ml LB medium in 14-ml test tubes and cultured at 37ºC and 250 rpm. At indicated time points, samples were collected for optical density (OD_{600}) measurements. n=4. Data corresponding to each time point represent mean value ± standard deviation.

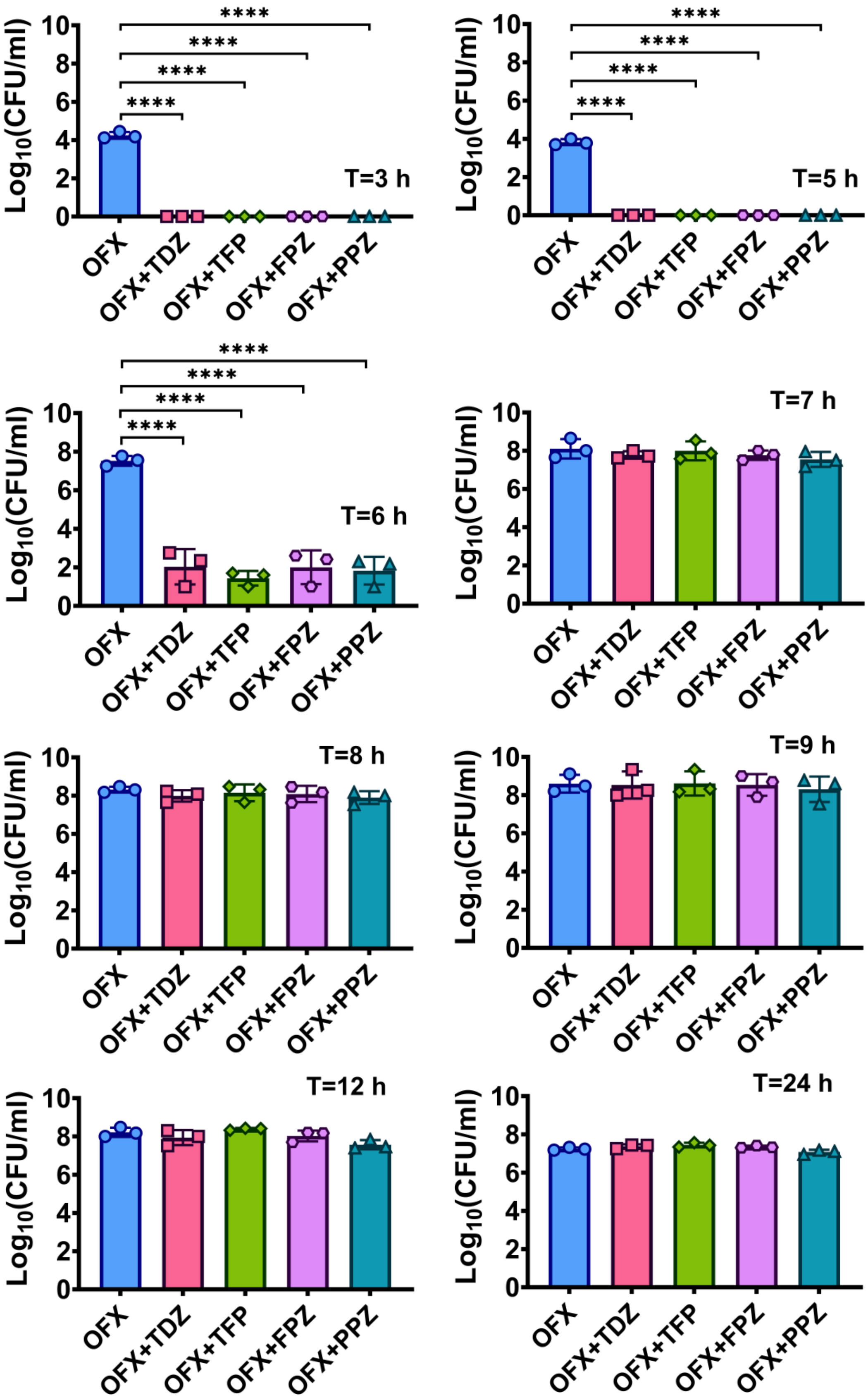
Log10(CFU/ml)
T=3 h
T=5 h
T=6 h
T=7 h
T=8 h
T=9 h
T=12 h
T=24 h
****
OFX
OFX+TDZ
OFX+TFP
OFX+FPZ
OFX+PPZ

Supplementary Fig. 12. Phenothiazine drugs reduce OFX persister levels in metabolically active cell populations. Cells at indicated time points of growth (see Supplementary **Fig. 1** for *E. coli* growth) were treated with OFX (5 µg/ml) and/or phenothiazines. After 20-h treatment, cells were plated for viable cell counts. TDZ: 0.25 mM; TFP: 0.5 mM; FPZ: 0.5 mM; PPZ: 0.5 mM. Statistical analysis was performed using one-way ANOVA with Dunnett's posttest. $****P < 0.0001$. n=3. Data corresponding to each time point represent mean value ± standard deviation.

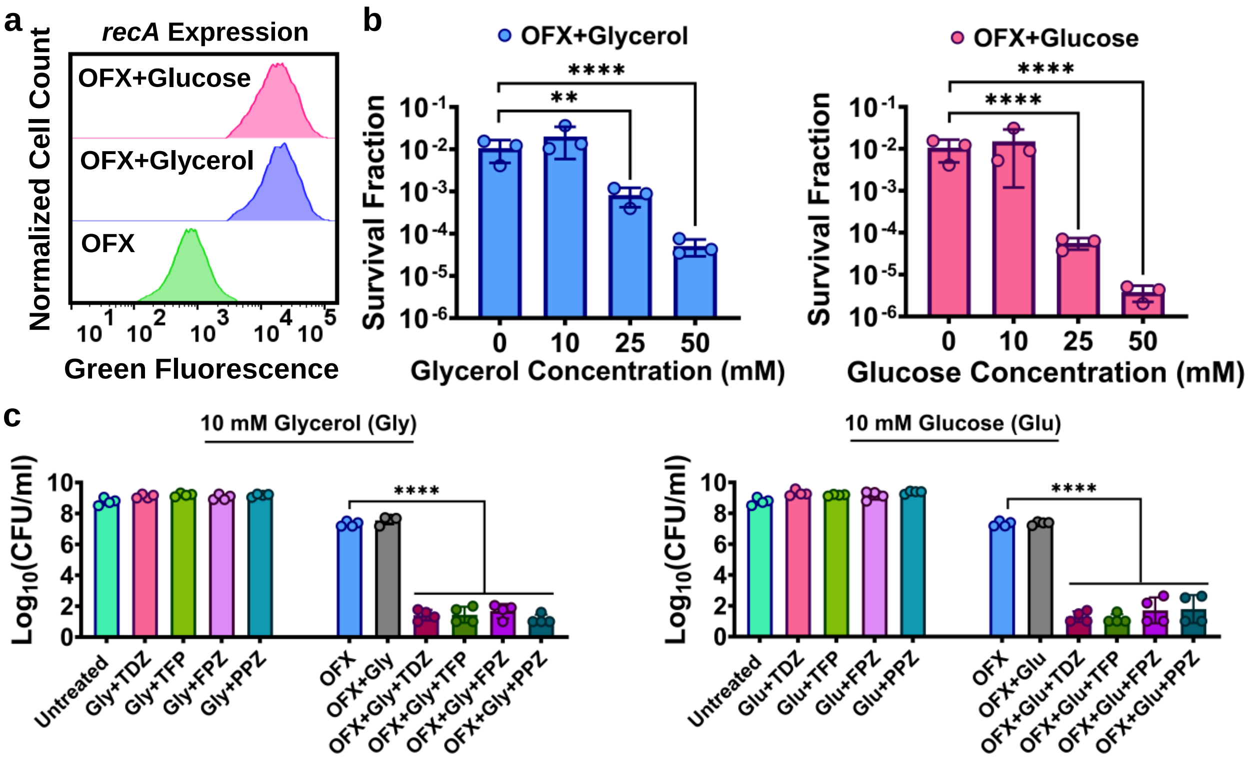

Supplementary Fig. 13. Metabolic Stimulation can potentiate OFX and phenothiazine activities in stationary phase *E. coli* MG1655 cells. (a) Stimulation of *recA* expression by carbon sources. *E. coli* MG1655 cells with P$_{recA}$-*gfp* were supplemented with glycerol (10 mM) or glucose (10 mM) at mid-stationary phase, and 30 min later, cells were treated with OFX (5 µg/ml). After 20 h of treatment, cells were diluted in PBS and analyzed with a flow cytometer. A representative flow diagrams is shown in the figure. All independent biological replicates have similar results. n=3. **(b)** Persister levels in metabolically stimulated cultures. Mid-stationary phase *E. coli* MG1655 cells were supplemented with glycerol or glucose at indicated concentrations, and 30 min later, cells were treated with OFX (5 µg/ml). After 20 h of treatment, cells were plated for viable cell counts. n=3. **(c)** Survived cell levels in metabolically stimulated co-treated cultures. Cells at late-stationary phase (t=24 h) were treated with (i) OFX (5 µg/ml), (ii) OFX+ phenothiazine drugs or (iii) phenothiazine drugs 30 min after adding glycerol or glucose to the cultures. After 20-h treatment, cells were plated for viable cell counts. n=4. TDZ: 0.25 mM; TFP:0.5 mM; FPZ:0.5 mM; PPZ:0.5 mM. Statistical analysis was performed between the control and treatment group using One-way ANOVA with Dunnett's posttest. ****P < 0.0001. Data corresponding to each time point represent mean value ± standard deviation.

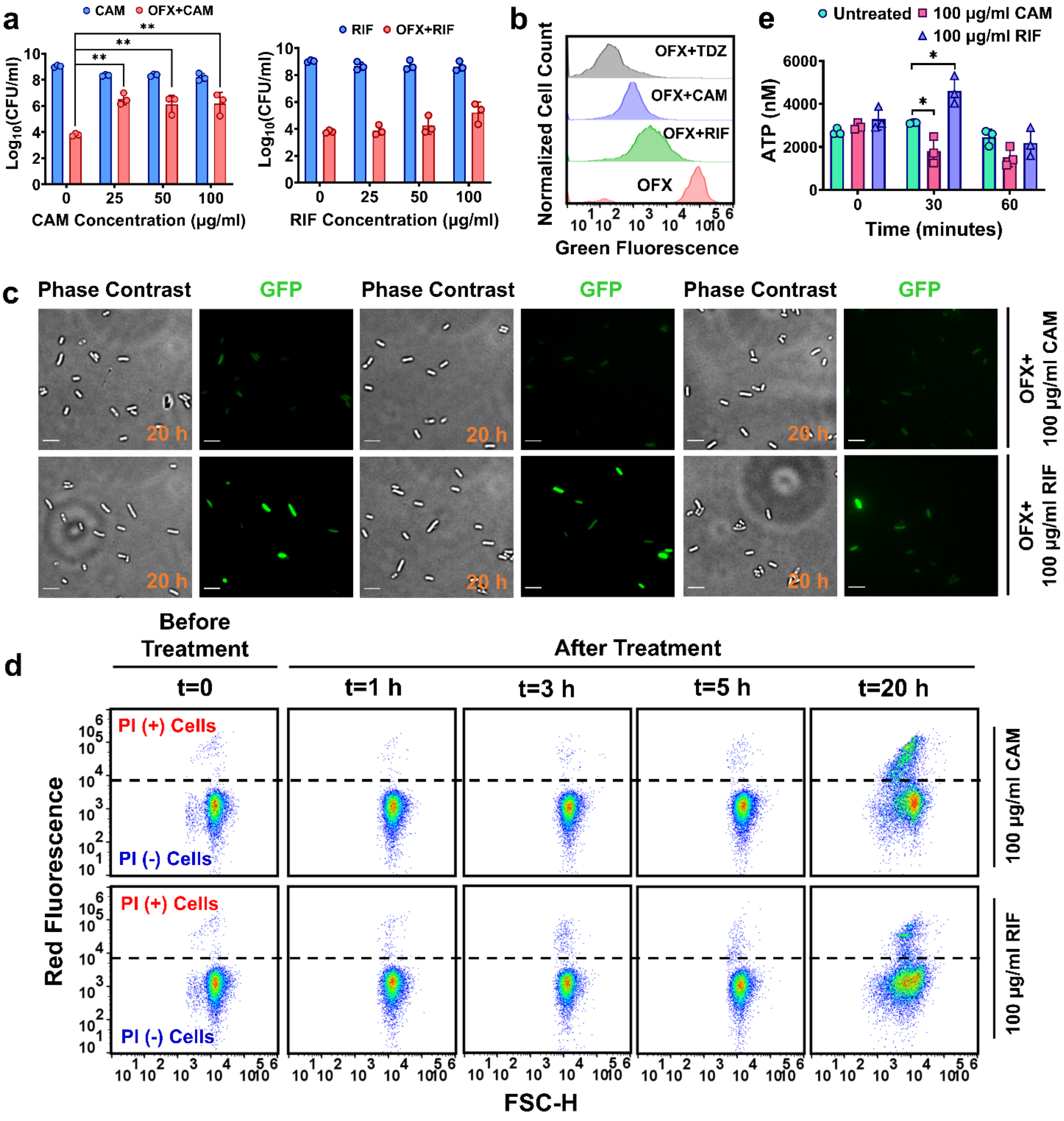

Supplementary Fig. 14. Chloramphenicol (CAM) and rifampicin (RIF) treatments do not eliminate persister cells in *E. coli* MG1655. (a) Cell survival after CAM or RIF treatment in the presence of OFX. Cells were treated with OFX (5 µg/ml) and CAM or RIF at indicated concentrations for 20 h, and then, plated for viable cell counts. n=3. **(b-c)** Inhibition of *recA* expression by CAM or RIF. GFP was measured with a flow cytometer and a microscope after cells at early stationary phase were treated with CAM or RIF at indicated concentrations in the presence of OFX (5 µg/ml) for 20 h. Cells treated only with OFX represent the positive control. A

representative flow cytometry diagram and microscope image are shown in the figure. All independent biological replicates have similar results. n=3. Scale bar: 5 µm. **(d)** Membrane integrity of CAM or RIF-treated cells. Cells at early-stationary phase were treated with the inhibitors at indicated concentrations. Cells before and after treatment were stained with PI at indicated time points for flow cytometry analysis. Live cells and ethanol-treated dead cells were used as negative and positive controls, respectively (see Supplementary **Fig. 8b**). A representative flow cytometry diagram is shown in the figure. All independent biological replicates have similar results. n=3. **(e)** ATP levels in CAM or RIF-treated cells. Early stationary phase cells were treated with the inhibitors at indicated concentrations, and 100 µl of cell cultures were collected to measure ATP concentrations 30 and 60 min later. n=3. Statistical analysis was performed between the control and treatment groups using One-way ANOVA with Dunnett's posttest. *P < 0.05, **P < 0.01. FSC-H: Forward Scatter. Data corresponding to each time point represent mean value ± standard deviation.

SUPPLEMENTARY TABLES

Supplementary Table 1. Concentrations of bactericidal antibiotics used in persister assays.

**Bacterial Strains**	**Bactericidal Antibiotics**	**MIC Range (µg/ml)**	**Persister Assay Concentration (µg/ml)**
Escherichia coli K-12 MG1655 Wild Type	Ofloxacin	0.039-0.078 (determined in our previous study[1])	5
Escherichia coli K-12 MG1655 Wild Type	Norfloxacin	0.064-0.094 (this study)	0.8
Escherichia coli K-12 MG1655 Wild Type	Moxifloxacin	0.064-0.094 (this study)	0.8
Escherichia coli K-12 MG1655 Wild Type	Levofloxacin	0.023-0.032 (this study)	0.3
Escherichia coli K-12 MG1655 Wild Type	Ciprofloxacin	0.023-0.032 (this study)	0.3
Escherichia coli K-12 MG1655 Wild Type	Ampicillin	3.125-6.25 (determined in our previous study[1])	200
Pseudomonas aeruginosa PA01	Ofloxacin	0.3125-0.625 (determined in our previous study[1])	5
Klebsiella pneumoniae CXY 130	Ofloxacin	2-3 (this study)	25
Acinetobacter baumannii BAA-1605	Ofloxacin	12-16 (this study)	140

Supplementary Table S2. Chemicals used in this study.

Chemicals	Purity (%)	Source	Catalog Number
Ofloxacin	98	Fisher Scientific	AC455670050
Norfloxacin	100	Fisher Scientific	ICN15594905
Moxifloxacin	≥97.5	Fisher Scientific	AC457960010
Levofloxacin	98	Fisher Scientific	AAJ6694306
Ciprofloxacin	98	Fisher Scientific	AC449620250
Ampicillin	>95	Fisher Scientific	BP1760-25
Kanamycin	>95	Fisher Scientific	AC450811000
Polymyxin B<br>(Potency: 7720.0 IU/mg)	N/A	Fisher Scientific	52-915-00MG
Chloramphenicol	98%	Fisher Scientific	AC227920250
Rifampicin	>98%	Fisher Scientific	R007925G
Isopropyl ß-D-1-thiogalactopyranoside	>99	Fisher Scientific	BP1755-10
Amitriptyline hydrochloride	≥98	VWR	50-144-4347
Trifluoperazine di-hydrochloride	≥98	Fisher Scientific	T28495G
Hexachlorophene	≥98	Fisher Scientific	M02191G
Thioridazine hydrochloride	>99	Fisher Scientific	30-705-0
Chlorpromazine hydrochloride	≥98	Fisher Scientific	C24815G
Pentachlorophenol	99.1	Fisher Scientific	NC1481997
Fluphenazine di-hydrochloride	≥98	Fisher Scientific	AAB2084814
Perphenazine	≥97	Fisher Scientific	P19705G
Potassium tellurite	98.2	VWR	IC10042725

Supplementary References

1. Mohiuddin, S. G., Hoang, T., Saba, A., Karki, P. & Orman, M. A. Identifying Metabolic Inhibitors to Reduce Bacterial Persistence. *Front. Microbiol.* **11**, 472 (2020).
