# Supplementary material for: Pleiotropic actions of phenothiazine drugs are detrimental to Gram-negative bacterial persister cells
Source: Commun Biol. 2022 Mar 9;5:217. doi: 10.1038/s42003-022-03172-8 (PMC8907348; doi:10.1038/s42003-022-03172-8)
Supplement: Supplementary file 3 — Description of Additional Supplementary Files [file 42003_2022_3172_MOESM3_ESM.pdf]

Description of Additional Supplementary Files

File name: Supplementary Data 1
Description: Bacterial strains and plasmids used in this study.

File name: Supplementary Data 2
Description: Oligonucleotides for the generation and verification of mutant strains.
